# On the Clinical Analysis of the Tennessee Collection of Urinary Calculi

**Published:** 1855-04

**Authors:** 


Art. VII.—*On the Clinical Analysis of the Tennessee Collection of Urinary Calculi;* by E. B. Haskins, M. D. A report read to the Tennessee Medical Society and published in the Transactions of the Society. Clarksville. 1855.

---

This report embraces the results of the analysis of one hundred and seventy-six urinary calculi, constituting what is called the Tennessee collection. Of this number one hundred and seven were duplicate concretions. Four calculi from the lower animals were examined which increases the whole number to one hundred and eighty. Subtract the duplicates and there remain seventy-three distinct calculi analysed, sixty-nine being from the human subject. The results obtained by Dr. Haskins are entirely in harmony with those of the Transylvania collection published by Dr. Peter.

In regard to the origin of urinary calculi, Dr. Haskins differs from the opinion which has prevailed to a considerable extent and which attributes the origin of a great majority of these bodies to general causes. He believes that very few, if any, are produced by general causes, whilst the great majority arise from local ones; "that the *latter* constitutes the law, and the *former* the exception."

To all persons interested in the subject of urinary deposits the tables of Dr. Haskins will afford much that is valuable, bearing especially upon the chemical constitution of these bodies. The analyses have been conducted with great care and minuteness and the results may be relied on as being entirely accurate and truthful. Dr. Haskins did not undertake the task with the view of answering any special question in either animal chemistry, physiology or ætiology, but simply to place on record additional facts, which now for the first time meet the public eye, whether they should answer *existing* questions or propound *new* ones. He has acquitted himself with his accustomed credit, and has eliminated from the materials which he collected a number of exceedingly interesting chemico-medical facts.

